# Fetal Sex and Fetal Environment Interact to Alter Diameter, Myogenic Tone, and Contractile Response to Thromboxane Analog in Rat Umbilical Cord Vessels

**DOI:** 10.3389/fphys.2021.620058

**Published:** 2021-09-16

**Authors:** Benoit Sicotte, Michèle Brochu

**Affiliations:** Department of Pharmacology and Physiology, Faculty of Medicine, Université de Montréal, Montreal, QC, Canada

**Keywords:** thromboxane analog, fetal adverse environment, rat, vascular function, umbilical cords

## Abstract

Fetal growth needs adequate blood perfusion from both sides of the placenta, on the maternal side through the uterine vessels and on the fetal side through the umbilical cord. In a model of intrauterine growth restriction (IUGR) induced by reduced blood volume expansion, uterine artery remodeling was blunted. The aim of this study is to determine if IUGR and fetus sex alter the functional and mechanical parameters of umbilical cord blood vessels. Pregnant rats were given a low sodium (IUGR) or a control diet for the last 7 days of pregnancy. Umbilical arteries and veins from term (22 day) fetal rats were isolated and set-up in wire myographs. Myogenic tone, diameter, length tension curve and contractile response to thromboxane analog U46619 and serotonin (5-HT) were measured. In arteries from IUGR fetuses, myogenic tone was increased in both sexes while diameter was significantly greater only in male fetuses. In umbilical arteries collected from the control group, the maximal contraction to U46619 was lower in females than males. Compared to the control groups, the maximal response decreased in IUGR male arteries and increased in female ones, thus abolishing the sexual dimorphism observed in the control groups. Reduced contractile response to U46619 was observed in the IUGR vein of both sexes. No difference between groups was observed in response to 5HT in arteries. In conclusion, the change in parameters of the umbilical cord blood vessels in response to a mild insult seems to show adaptation that favors better exchange of deoxygenated and wasted blood from the fetus to the placenta with increased myogenic tone.

## Introduction

Adequate blood perfusion from both sides of the placental barrier is a prerequisite to the successful growth of the fetus. The umbilical veins carry nutrient and oxygen from the placenta to the fetus as the umbilical arteries carry waste products from the fetal circulation to the mother. In rats, differentiation of umbilical blood vessels occurs between 17 and 21 days; mesenchymal cells differentiate into mature smooth muscle cells and endothelium becomes attenuated (Leeson and Leeson, 1965; Arishima et al., 1990). As the umbilical cord lacks innervations, its vascular tone depends of the viscoelastic properties and is under the control of vasoactive substances within the circulation, released locally within the vessel wall or from the placenta.

Abnormal Doppler waveforms are observed in the umbilical arteries of intrauterine growth restricted (IUGR) fetuses. This is associated with an increased placental vascular resistance (Kalache and Duckelmann, 2012), which might be caused by structural modifications to the placental microcirculatory system, by an increased thickness of stem villi vessel walls and a decreased in lumen circumference (Mitra et al., 2000) or by an abnormal number of tortuous vessels (Baykal et al., 2004). However, increased stiffness of the umbilical artery could also induce an abnormal Doppler velocimetry, with a blood flow that is more pulsatile and intermittent leading to an inadequate nutrient exchange, as previously shown in sheep (Dodson et al., 2013) and humans (Burkhardt et al., 2009). The vasoactive responses of arteries in IUGR animals could be altered in response to placental insufficiency. Indeed, the contractile response to KCl was decreased in umbilical arteries from IUGR guinea pigs compared to their control group counterparts (Canas et al., 2017).

The umbilical venous cross-sectional area is inversely correlated to the degree of umbilical ring constriction in the abdominal wall in human male fetuses (Skulstad et al., 2006). During gestation weeks 20–36, uterine artery Doppler indexes are higher in women bearing female fetuses compared to ones with male fetuses (Widnes et al., 2018). The signaling pathways in the autophagic process induced by starvation are different in smooth muscle cells obtained from male human umbilical artery than from female ones (Campesi et al., 2016). Recently, Balzano et al. (2019) found that stem cells isolated from Wharton’s jelly from female fetuses showed different gene expression than male ones. All these results suggest that fetal sex is of importance when studying the umbilical cord. However, to our knowledge, sex differences in umbilical vessel properties are not documented.

We developed an IUGR rat model by giving a low sodium diet to dams during the last week of gestation (Roy-Clavel et al., 1999; Battista et al., 2002; Bedard et al., 2005; Bibeau et al., 2010; Bigonnesse et al., 2018). This treatment reduces normal maternal plasma volume expansion (Roy-Clavel et al., 1999) and leads to a decreased diameter of uterine arcuate and radial arteries, as well as to placental hypoxia (St-Louis et al., 2006; Bibeau et al., 2016), compared to the control group. An impaired blood velocity and an increased resistance index in the main uterine artery were also reported (Bibeau et al., 2016) as well as an enhanced reactivity to Angiotensin II and an increased myogenic tone in the arcuate and radial uterine arteries from this model (St-Louis et al., 2006; Bibeau et al., 2016). Myogenic tone is a property of small vessels that contract in response to increased transmural pressure and dilate with decreased pressure (Davis and Hill, 1999). In isometric preparations, vascular myogenic response is represented by a secondary increase in tension in response to stretch activation (Davis and Hill, 1999). Myogenic tone is obtained by the difference between stretch-tension curve in presence of Ca^++^ and the one obtained in Ca^++^-free bathing solution.

In view of these findings, we hypothesized that, in response to decreased placental perfusion induced by low sodium diet, the tonus and reactivity of the umbilical cord blood vessels will be increased, in a sex-specific manner, to provide better placental exchange. Thus, myogenic tone, diameter, passive mechanical properties of term umbilical arteries and veins and their contractile response to a thromboxane A2 mimetic U46619 and to serotonin (5-HT) were measured in wire myographs.

## Materials and Methods

### Animals

This study was carried out in strict accordance with the recommendations of the Canadian Council on Animal Care. The experimental procedures were approved by the Animal Care Committee of the Université de Montréal. Female Sprague-Dawley rats (Charles River Canada, Saint-Constant, QC, Canada) weighing 225–250 g were mated with a known fertile male. Day 1 of pregnancy was determined by the presence of spermatozoa in morning vaginal smears. All animals were housed under controlled lighting (6 AM–6 PM) and temperature (21 ± 3°C). The dams were randomly assigned to 1 of 2 *ad libitum* diets for the last 7 days of gestation (term = day 23). The control group was fed a normal diet containing 0.20% sodium and 0.40% potassium (normal diet 5755; PMI Feed Inc., Ren’s Feed and Supplies, Oakville, ON, Canada) and tap water. The experimental group, IUGR, as previously described (Roy-Clavel et al., 1999; Battista et al., 2002; Bedard et al., 2005; Bibeau et al., 2010; Bigonnesse et al., 2018) received a low-sodium diet containing 0.03% sodium and 0.85% potassium (low-sodium diet 5881; PMI Feed Inc.) and demineralized water. The composition of both control and experimental diets was similar in protein (19%), carbohydrate (60.6%), and fat (10%) content. On day 22 of gestation, rats were anesthetized in an induction chamber with 4% isoflurane in O_2_ using a Dispomed vaporizer. Anesthesia was considered completed when rats were sleeping on their side breathing normally and without reaction when pinched on the paw. They were then sacrificed by decapitation. Uteri were excised, opened longitudinally and fetuses with their placentae were removed, sexed, and placed on a heating pad. Male and female placentae with umbilical cord were collected and immersed in an ice cold physiological salt solution (PSS) of the following composition in mM: NaCl 118; KCl 4.65; CaCl_2_ 2.5; MgSO_4_ 1.18; KH_2_PO_4_ 1.18; NaHCO_3_ 25; and dextrose 5.55. Fetuses were sacrificed by decapitation thereafter. The control and IUGR umbilical cords were separated into male and female sub-groups.

### Preparation of Umbilical Cord Vessels

**FIGURE 1:**
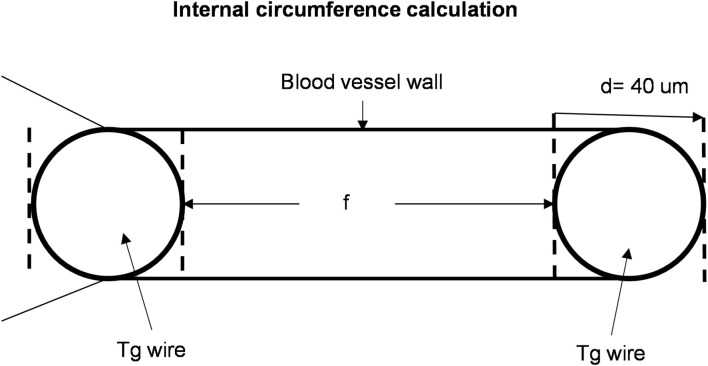
Measurement of the internal circumference. f, distance in μm between the inner edges of the wires; tg wire, tungsten wire; d, diameter of the wire. Internal circumference (L) is given by: L = (π + 2)d + 2f.

The placenta with its umbilical cord was pinned down in a petri dish filled with SYLGARD 184 silicone containing cold PSS. Both vessels, artery and vein, run parallel in the cord. The umbilical artery and vein were dissected from surrounding connective tissue in the medial section of the cord under a stereo-microscope and cut in 2 mm ring segments. For each experiment, one type of vessel, artery or vein, was isolated but not both. Two 40 μm tungsten wires were inserted through the lumen of the ring segment and were installed on the two myograph (Kent Scientific Corporation, TIS8105R) jaws. One was attached to an isometric force transducer and the other to a micrometer device for adjustment of the vessel segment internal circumference (L) that was calculated from L = (π + 2)d + 2f where d is the wires diameter and f the distance between the inner edges of the wires (Figure 1; Mulvany and Halpern, 1977; Angus and Wright, 2000). The vessel segment was bathed in a PSS maintained at 37° and bubbled with a mixture of 95% O_2_ and 5% CO_2_, pH 7.4 and equilibrated for 30 min before experiments.

### Determination of Optimal Internal Circumference

**FIGURE 2:**
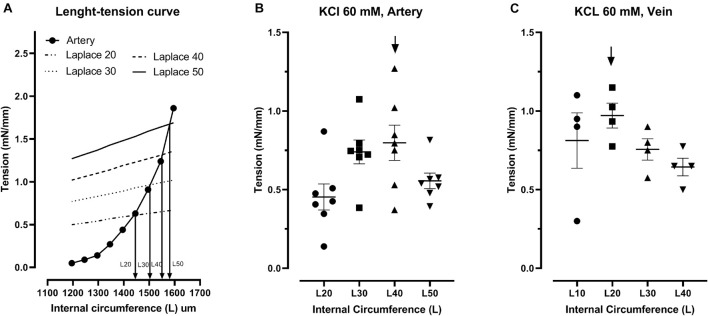
Determination of optimal internal circumference. Umbilical artery length-tension curve and theoretical straight line of Laplace (for a pressure of 20, 30, 40, and 50 mmHg) **(A)**. Tension developed in response to 60 mM KCl at different internal circumference in arteries *n* = 8 **(B)** and veins *n* = 4 **(C)**. Arrow indicated the optimal internal circumference for both vessels in subsequent experiments.

The resting wall stretch influences *in vitro* vascular contractile response to pharmacological agents (Sparks and Jr Bohr, 1962; Dobrin, 1978). Therefore, preliminary experiments were done to determine the optimal internal circumference (L) at which vessels generated maximal active force in response to a contractile agent. First, the two wires were brought up to touch each other. Micrometer device was set to zero. The wires were then moved apart until a very small tension was sensed by the force transducer. The distance indicated by the micrometer was recorded and internal circumference zero (L0) was calculated. Umbilical vessels were then stretched using the micrometer by steps of 50 μm until 300–350 μm for the artery and by 100 μm steps reaching 700–800 μm for the vein. Tension was recorded at each step to obtain an exponential length-tension (L-T) curve. The intersection of this curve with the straight line of Laplace equation (T = r_*i*_P where r_*i*_ is internal radius and P the transmural pressure) will give the internal circumference (L) for a given transmural pressure (Figure 2A; St-Louis et al., 2006). Umbilical arteries were set from L20 to L50 (L20: internal circumference for which the transmural pressure was 20 mmHg) and umbilical veins from L10 to L40. Then, the vessels were challenged with 60 mM potassium chloride in PSS at these different internal circumferences. The optimal internal circumference for umbilical arteries (*n* = 7 fetuses) and veins (*n* = 4 fetuses) were L40 and L20, respectively (Figures 2B,C). Vessels were then set at these lengths for subsequent experiments.

### Experimental Protocol

**FIGURE 3:**
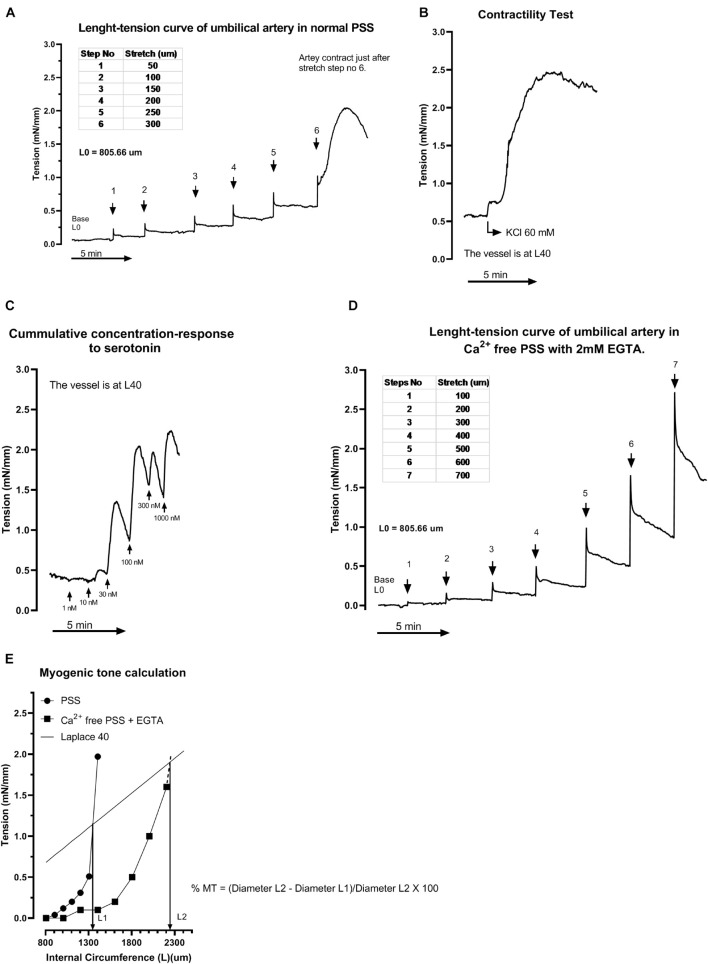
Representative tracing of the experimental protocol in an umbilical artery (similar protocol for the vein). Vessels were set at L0, a first length-tension (L-T) curve is recorded to normalize the vessels at L40 or L20 **(A)**. Then, contractility of the preparation is tested with a 60 mM potassium chloride challenge **(B)**. PSS is replaced by fresh PSS and vessels are allowed to rest 60 min before cumulative concentration-response curve to U46619 or Serotonin **(C)**. After washing the contractile agent response with fresh PSS, Ca^2+^ free PSS (containing 2 mM EGTA) is added. The vessels are returned to L0, second L-T curve is measured with stretch steps of 100 μm **(D)**. L40 or L20 are recorded and diameter in free calcium PSS calculated and recorded. Myogenic tone is calculated using this formula: % MT for artery = (Diameter L40 in Ca^2+^ free PSS – Diameter L40 in PSS)/Diameter L40 Ca^2+^ free PSS × 100 **(E)**.

**FIGURE 4:**
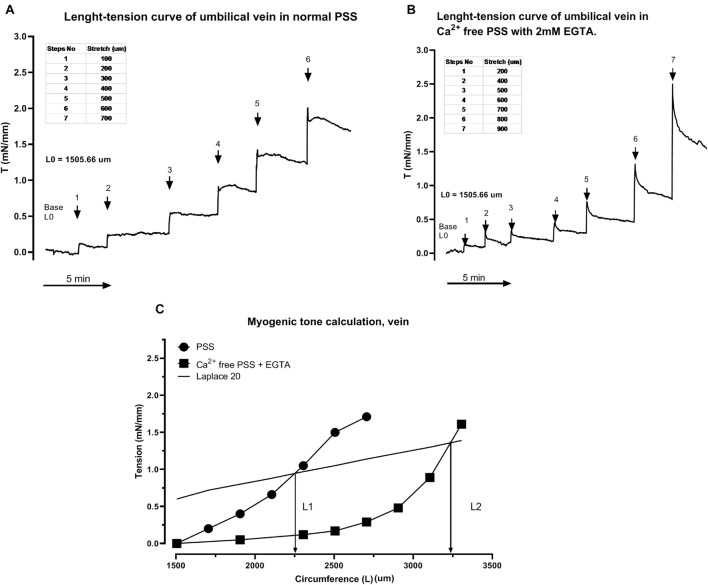
Representative tracing of the experimental protocol in an umbilical vein for myogenic tone measurement. Length-tension (L-T) curve in PSS **(A)**. Length-tension curve in Ca^2+^ free PSS **(B)**. Determination of myogenic tone **(C)**.

Following installation in a myograph and equilibration, initial internal circumference (L0) was determined. Thereafter, the L-T curve was performed to normalize the vessel (Figure 3A artery and Figure 4A, vein) and determine the internal circumference under a transmural pressure of 40 mmHg for the artery (L40) and 20 for the vein (L20) using Laplace equation (as in Figure 2A). The contractility of the segments was confirmed with a 60 mM potassium chloride (KCl) challenge, non-responsive segments were discarded (Figure 3B). After a 60 min resting period, the cumulative concentration-response curve to 9,11-dideoxy-9α,11α-methanoepoxy PGF_2α_, U46619, a thromboxane A_2_ analog, was constructed (Figure 3C). Segments were then washed until a basal tension level was reached. The PSS was then replaced with a free calcium PSS containing 2 mM EGTA (Ca^2+^ free PSS), the vessel segments were returned to L0 and new L-T curves were performed to obtain L40 and L20 for artery an vein respectively (Figure 3D, artery and Figure 4B, vein). This second L-T curve was right shifted (Figure 3E, artery and Figure 4C, vein) compared to the one in normal PSS, indicating a myogenic tone of the vessel in normal PSS. The umbilical vessel myogenic tone was calculated in % using diameter at L40 (artery) or L20 (vein) in a Ca^2+^ free PSS and in a normal PSS [% MT for artery = (Diameter L40 in Ca^2+^ free PSS − Diameter L40 in PSS)/Diameter L40 Ca^2+^ free PSS × 100) (Figure 3E artery and Figure 4C, vein). Diameter and stiffness (passive mechanical property depending of the structural elements such as elastin and collagen within the vessel wall) of the vessels were compared using L-T curves in Ca^2+^ free PSS to avoid influence of smooth muscle contractility. A second set of experiments were conducted on arteries using serotonin as a contractile agent.

### Statistical Analysis

Internal circumferences in Ca^2+^ free PSS L-T curves were transformed by L/L0. Data were analyzed by computer fitting to an exponential equation, y = A e^*B*(*L*/L0)^, in which A is the tension at L0, L/L0 is the relative change in circumference of the vessel segments, and B is the changes in relative circumference required to modify the resting tension and is used as an estimate of vessel stiffness. Diameter, B parameter and myogenic tone results were tested for normality using the d’Agostino and Pearson normality test. The concentration-response curves were fitted to a four parameters logistic equation to evaluate the maximum response (E_*max*_). B parameter, Emax, passive diameter, and myogenic tone were compared by two-way analysis of variance, with sex and IUGR as factors, followed by a Bonferroni multiple comparison posttest. Values were considered statistically significant when they reached at least *p* < 0.05. Data are reported as means ± SEM along with the best fitted curve to the data points, *n* representing the number of animals from different litters. The software Graphpad PRISM V4 was used to analyze the data.

## Results

### Mechanical Properties of Umbilical Vessels

**FIGURE 5:**
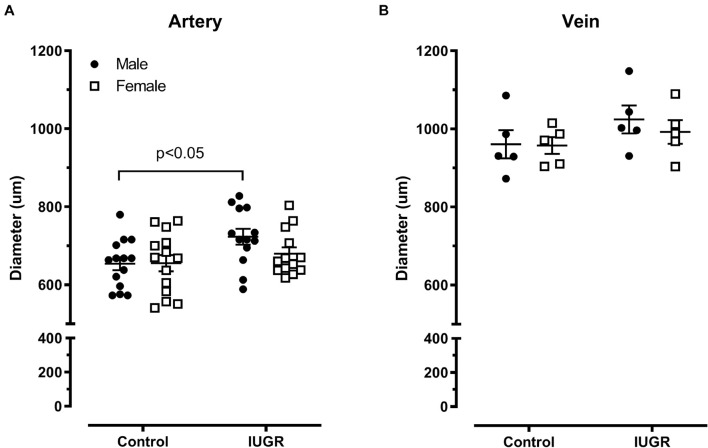
Umbilical artery **(A)** and vein **(B)** passive diameter in micrometer (μm) for the different groups of animals. Control (*n* = 14), IUGR (*n* = 13). Lines represents mean ± SEM.

The umbilical artery diameter was increased in the IUGR groups (*p* < 0.05, two-way ANOVA) and this reached statistical significance for males (*p* < 0.05, Bonferroni test). The diameters were similar between male and female in the control group (Figure 5A). As expected, the diameter of the umbilical vein is greater than that of the artery (Figure 5B). The sex of the fetus or pregnancy conditions did not affect this parameter.

**FIGURE 6:**
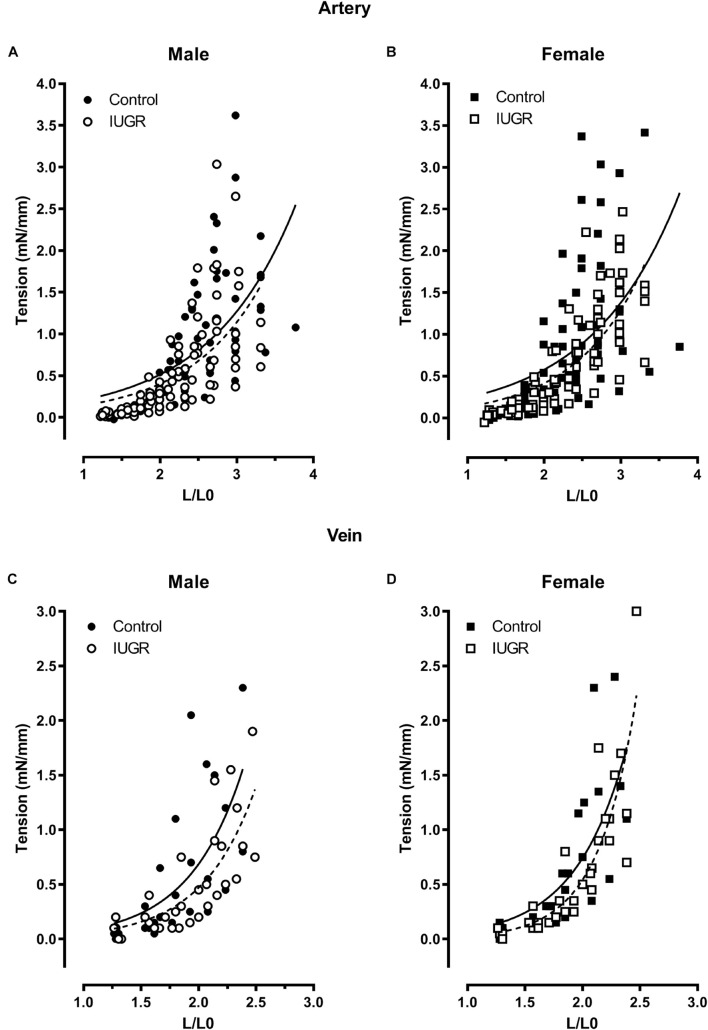
Length-tension relationship in the umbilical artery (**A,B**, control *n* = 14, IUGR *n* = 13) and vein (**C,D**, *n* = 5, each group). The lines are the best fit of all experimental points (solid: control; dotted: IUGR).

Figure 6 depicts the L-T relationship in umbilical vessels. No differences were observed between vessels from IUGR and control group males (Figures 6A,C) nor between the females from both groups (Figures 6B,D). The stiffness of the vessel was estimated by the exponential B parameter of the curve (Table 1). No differences were observed between sexes or between the IUGR and control groups. These results indicate that fetal condition and sex did not alter the wall components of the vessels. The B parameter for the vein was greater than for the artery, indicating a more rigid blood vessel wall (Table 1).

**TABLE 1:** Stiffness (B) parameter of artery and vein from control and IUGR fetuses.

	Artery		Two-way ANOVA		
	Ctl	IUGR	Sex	IUGR	Interaction
	*n* = 14	*n* = 13			
Male	1.65 ± 0.15	1.71 ± 0.20	NS	NS	NS
Female	1.62 ± 0.18	1.69 ± 0.12			

	**Vein**		**Two-way ANOVA**		
	**CtlL**	**IUGR**	**Sex**	**IUGR**	**Interaction**
	***n* = 5**	***n* = 5**			

Male	2.26 ± 0.52	2.95 ± 0.50	NS	NS	NS
Female	2.92 ± 0.46	2.61 ± 0.56			

*Comparison by two-way analysis of variance, with sex and IUGR as factors, followed by a Bonferroni multiple comparison posttest.*

### Contractile Responses in Umbilical Vessels

**FIGURE 7:**
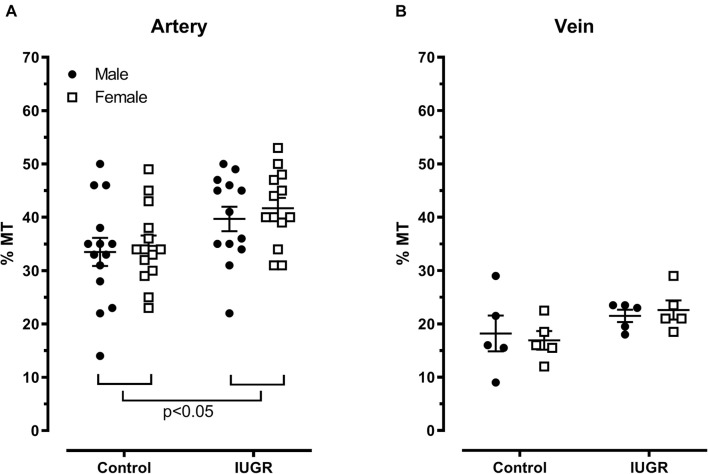
Percent myogenic tone (%MT) in the umbilical artery **(A)** and vein **(B)**. Control (*n* = 14), IUGR (*n* = 13). Lines represents mean ± SEM.

The percentage of myogenic tone is increased in umbilical arteries from the IUGR group compared to their control group counterparts (Figure 7A, *p* < 0.05, two-way ANOVA) without sex effect. The umbilical vein myogenic response is lower than in arteries but does not differ between the four groups (Figure 7B).

**FIGURE 8:**
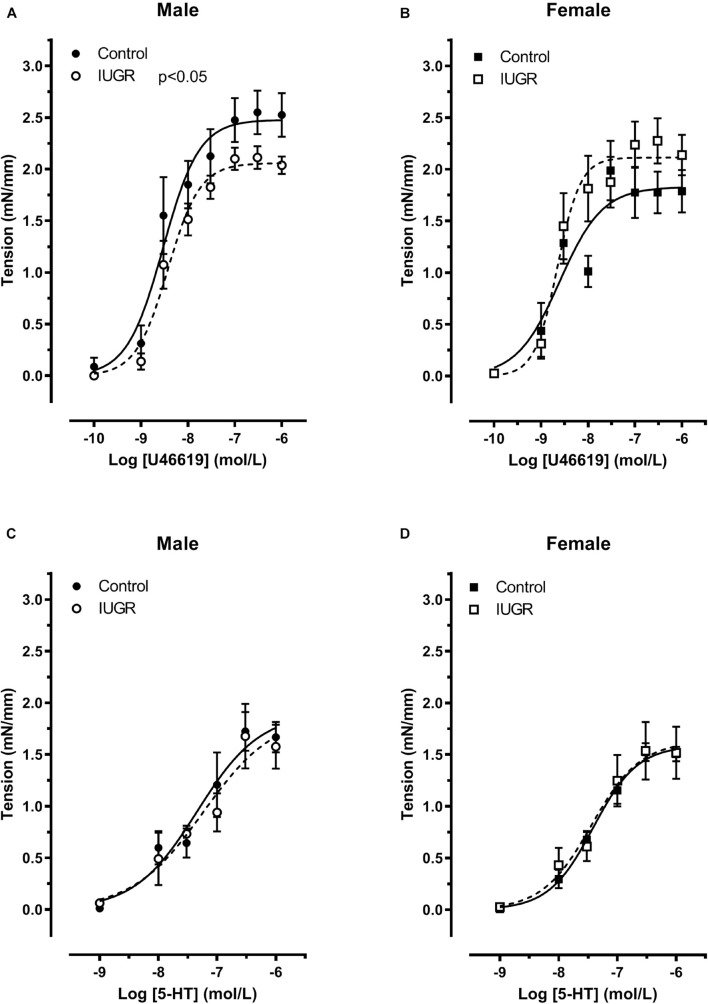
Contractile responses to U46619 (**A,B**, *n* = 8) and serotonin (5-HT) (**C,D**, control *n* = 6, IUGR *n* = 5) in umbilical artery of control and IUGR fetuses. Each points is the mean ± SEM of *n* animals for each groups and sexes. The lines represents the best fit of all experimental points.

In order to determine the response of the vessels to vasoconstrictors, dose-response curves to U46619 and to serotonin (5-HT) were done. Figure 8 depicts myotropic responses of umbilical artery from control and IUGR rat fetuses. There is an interaction between sex and IUGR (*p* < 0.05, two-way ANOVA). Indeed, the decreased response to U46619 is observed in umbilical arteries from male IUGR compared to their control group counterparts (Figure 8A, Emax 2.06 ± 0.08 vs 2.48 ± 0.14 mN/mm, respectively, *p* < 0.05, Bonferroni test), while there is an increase in the IUGR female group that however did not reach statistical significance (Figure 8B, Emax 1.83 ± 0.14 vs 2.11 ± 0.11 mN/mm, respectively). Moreover, in control groups, female response is decreased compared to male (*p* < 0.05, two-way ANOVA). Both factors, IUGR and sex, did not affect contractile response to serotonin (Figures 8C,D).

**FIGURE 9:**
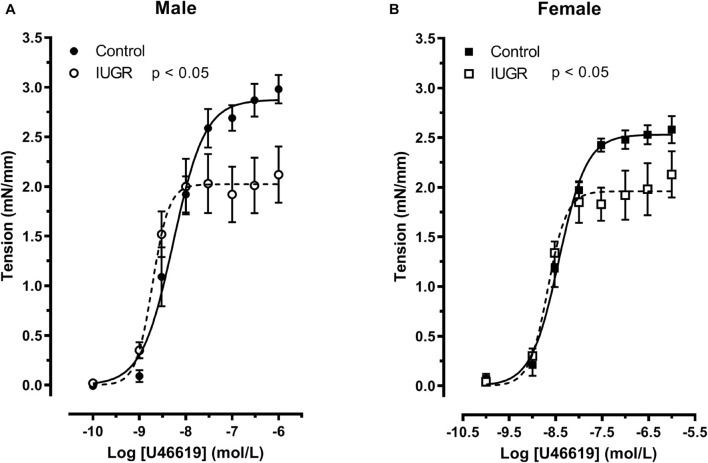
Contractile responses to U46619 in umbilical vein of male **(A)** and female **(B)** fetuses. Each points is the mean ± SEM of animals (*n* = 5) for each groups and sexes. The lines represents the best fit of all experimental points.

The responses to U46619 in umbilical veins are shown in Figure 9. IUGR shows markedly reduced maximal contraction to U46619 in both sexes (*p* < 0.05, two-way ANOVA, Bonferroni test). As observed in umbilical arteries, the female control group vessel contraction is reduced compared to male (*p* < 0.05, two-way ANOVA).

## Discussion

In the present study, we showed that sex and fetal environment alter the diameter of umbilical cord vessels and the contractile response to an analog of Thromboxane A2, which could lead to an adaptation during IUGR.

Using high resolution X-ray micro-computed tomography, Rennie et al. (2017) analyzed arterial and venous fetoplacental vasculature in E15.5 mice. By using computational flow modeling, they determined that the umbilical artery and vein represent respectively, 13 and 11% of the total fetoplacental vascular resistance. To our knowledge, these analyses were not done in rats, but we could however speculate that it would be similar. Considering that 24% of the fetoplacental vascular resistance comes from the umbilical vessels, their mechanical and vasoactive properties are of interest to estimate placental perfusion from the fetal side and ultimately the oxygen and nutriments exchange capacity.

### Increased Diameter of Umbilical Arteries From IUGR Rats

In the present study, the umbilical veins were larger in diameter than the umbilical arteries, that was not the case in the study by Rennie et al. (2017) where the umbilical vein and the artery diameter were similar. This discrepancy with our results could be explained by the species and by the gestational age, E15.5 in their study and term-pregnancy E22 in ours. Indeed, rat fetuses have two umbilical arteries and one vein until E14, with both arteries having a caliber of 120 μm. Then, the left umbilical artery reduces in caliber and by E17, it is completely closed. Using frozen fetuses under a dissecting microscope, Arishima et al. (1990) observed that the caliber of the right umbilical artery increases as pregnancy progresses to reach approximatively 450 μm at 21 days. We obtained a diameter of 655 μm for control group fetuses on fresh vessels mounted on a myograph under a passive tension of 40 mmHg in calcium free PSS, which produces a passive diameter reading. The technique used could explained the slight difference in diameters between both studies.

Umbilical artery diameter was significantly increased in IUGR fetuses and more specifically in males, indicating a greater capacity of the umbilical artery to transport deoxygenated and nutrient depleted blood to the placenta. Neither fetal environment nor sex had an effect on the umbilical vein diameter. Viscoelastic property did not differ in the different groups (sex or IUGR) in the two cord blood vessels, as shown by the L-T curve B parameter comparison. The umbilical vein had higher stiffness than the artery meaning that for a similar increase in length, more tension is developed by the vein. This result is consistent with those obtained with rat aorta and vena cava (Kalache and Duckelmann, 2012). Increased stiffness of umbilical arteries from infants with IUGR (Burkhardt et al., 2009) and from lamb in a sheep model of placental insufficiency were reported (Dodson et al., 2013). The discrepancy with the present study could be explained by the type and the duration of the prenatal insult. In the sheep model, the exposure to elevated temperature from E35 to E115 day for a 148 day term could lead to a remodeling of the umbilical arteries, which is not the case in the present model. There is a 36% decrease in fetal weight for both studies and 51% decrease in placental weight for the sheep model, which is more severe than in the rat model used herein [15–20% in fetal weight and ∼12% in placental weight (Roy-Clavel et al., 1999; Battista et al., 2002; Bedard et al., 2005; Bibeau et al., 2010; Bigonnesse et al., 2018)]. Using a mice model of fetal growth restriction induced by a combination antiretroviral therapy (cART), Cahill et al. (2019) showed by an ultrasound technique that the thickness and the stiffness of umbilical artery vessel wall at E17.5 were increased in the IUGR group; no difference were seen in the diameter of this artery between IUGR and control groups. Considering sexual dimorphism in IUGR artery diameter observed in our study, it is possible that the absence of difference could be hidden by the absence of sex determination in their experiment (Cahill et al., 2019).

In normotensive rats, increased blood flow induced by selective ligation of arteries caused an increase in lumen size without change of the structural composition of the vessel wall (Gao et al., 2008). During normal pregnancy, uterine radial arteries undergo outward expansive remodeling (increased lumen diameter and unchanged wall thickness) in order to reduce uterine vascular resistance. The mechanisms that induce this remodeling are increased wall shear stress, endothelial NO release and vasoactive and growth factors secreted by the placenta (for a review, Osol et al., 2019). In the present model of IUGR, an increase in the brain-to-body weight and ventricle-to-body weight ratios suggest a redistribution of cardiac output (Battista et al., 2002). It is possible that increased blood flow to the umbilical artery results in a greater umbilical artery diameter in the IUGR groups. This reaches significance only in male probably in response to factors secreted by the placenta. Indeed, sex- and diet-specific gene expression patterns were observed in the placentae of male and female mouse fetuses (Gabory et al., 2012).

### Increased Myogenic Tone of Umbilical Vessels From IUGR Rats

Myogenic tone is the ability of small vessels to contract or dilate in response to increased or decreased internal pressure. It plays a role in the blood flow autoregulation and the capillary pressure regulation to avoid fluid leakage and tissue damage. *In vivo*, autoregulation is achieved by myogenic response and metabolic control mechanisms (Davis and Hill, 1999; Davis, 2012; Kauffenstein et al., 2012). In human and in rodents, myogenic tone of uterine arteries appears in late pregnancy (Veerareddy et al., 2004; Telezhkin et al., 2008; Gokina et al., 2009). In the present model, we showed that both types of umbilical blood vessels exhibited myogenic tone in response to a circumferential stretch, an observation reported for the first time in rats. This is a surprising observation in such large diameter blood vessels, myogenic responsiveness is mostly observed in small diameter (<300 μm) blood vessels (Sun et al., 1992; Davis, 1993; Davis and Hill, 1999). Mesenteric arteries of 300 μm did not show any myogenic response in a pressurized myograph (Sun et al., 1992). Why would the umbilical vessels have myogenic tone? It could be the type of vessel and the time of pregnancy; umbilical vessels undergo vasoconstrictive closure at birth or when exposed to the extra-uterine environment. Another explanation is a better control of the local blood flow to avoid vasculature damage in the arterial tree of the fetoplacental unit, to maintain a constant perfusion and to promote a better exchange. The blood flow from or to the placenta in the umbilical cord vessels should be regulated to increase the capillary transit time to allow adequate gas, nutrient and waste exchange with maternal circulation. This will be accomplished by the interaction between myogenic tone and metabolic control mechanisms. We observed that the vein showed lower myogenic tone than the artery. In the arterial tree of fetoplacental unit, diameter vessels are smaller than in the venous tree (Rennie et al., 2017). The umbilical vein carries blood from the placenta to the portal system of the fetus and most of the blood bypasses the liver through the ductus venosus which enters the inferior vena cava. Thus, the greater myogenic tone in the umbilical artery would protect capillaries of the placenta from excessive blood flow.

Greater myogenic constriction was observed in IUGR arteries regardless of sex of the fetus. The mechanisms underlying the myogenic response have been studied for the last 20 years (Davis and Hill, 1999; Loufrani et al., 1999; Osol et al., 2002; Hill et al., 2009; Kauffenstein et al., 2012; Jackson, 2017; Dopico et al., 2018). Many mechanisms could be implicated in the increased myogenic tone in umbilical arteries from the IUGR model. Placentas were smaller; they displayed increased expression of hypoxia markers and increased glycogen cells (Bibeau et al., 2016). Thus, the metabolic control could interfere with the myogenic tone to increase the capillary transit time in the fetoplacental unit. Another explanation could be the decreased serum sodium levels observed in males and females fetuses IUGR (Bibeau et al., 2010). Using rabbit facial vein, Henrion et al. (1997) showed that decreased extracellular sodium concentration augmented the myogenic tone. Finally, the role of aldosterone cannot be excluded since the fetal serum concentration in IUGR is elevated (Bibeau et al., 2010) and this hormone could be in part responsible for myogenic tone (Gorini et al., 2019).

### Fetal Sex Alters the Response to Thromboxane Analog in Umbilical Vessels From IUGR Rats

The Thromboxane A2 mimetic, U46619, induced contraction of the umbilical cord blood vessels with an EC_50_ in the nanomolar range, like in mice (Kusinski et al., 2009) and humans (Hausermann and St-Louis, 2011). The maximum contraction was considerably higher in control male umbilical arteries compared to the control female group. This confirms the sexual dimorphism and the importance of determining fetus sex in experiments. This effect is lost in IUGR, as male and female share the same maximal contraction in response to U46619. Indeed, the statistical analysis showed an interaction between sex and fetal environment, with a decrease in the maximal response to U46619 in IUGR male fetuses and a slight but non-significant increase in IUGR female fetuses, compared to their respective control groups. The umbilical vein showed the same pattern of reactivity to U46619 in the control group vessels, with females having a smaller maximal contraction than males, an effect absent in the IUGR group. However, the response to an adverse fetal environment is different, with a significant reduction in contractility observed in both sexes. Since there is no difference in contractility in response to serotonin between the four groups, it seems specific to the Thromboxane receptor or its intracellular signaling pathway.

Our hypothesis is not completely confirmed, the myogenic tone was increased in umbilical arteries from IUGR fetus of both sex but reactivity to Thromboxane analog is decreased in male but not in female. In conclusion, the change in parameters of the umbilical cord blood vessels in response to a mild insult seems to show adaptation that favors better exchange of deoxygenated and wasted blood from the fetus to the placenta with increased myogenic tone. Further experiments on a mechanistic standpoint would contribute to understand the sex effect on the contractile response to agonist.

## Data Availability Statement

The original contributions presented in the study are included in the article/supplementary material, further inquiries can be directed to the corresponding author/s.

## Ethics Statement

The animal study was reviewed and approved by the Animal Care Committee of the Université de Montréal, accredited by the Canadian Council on Animal Care.

## Author Contributions

BS designed and performed the experiments, analyzed the data, and wrote the manuscript. MB provided the funding, analyzed the data, and revised and edited the manuscript. Both authors contributed to the article and approved the submitted version.

## Conflict of Interest

The authors declare that the research was conducted in the absence of any commercial or financial relationships that could be construed as a potential conflict of interest.

## Publisher’s Note

All claims expressed in this article are solely those of the authors and do not necessarily represent those of their affiliated organizations, or those of the publisher, the editors and the reviewers. Any product that may be evaluated in this article, or claim that may be made by its manufacturer, is not guaranteed or endorsed by the publisher.
